# Aging, Wealth, and Prosociality: Insights From Large Nationally Representative Surveys

**DOI:** 10.1093/geroni/igaf122.3883

**Published:** 2025-12-31

**Authors:** Jack Kirwan, Enna Chen, Laura Carstensen

**Affiliations:** Stanford Center on Longevity, Stanford, California, United States; Stanford University, Stanford, California, United States; Stanford University, Stanford, California, United States

## Abstract

Socioemotional selectivity theory (SST) posits that as future time horizons shorten, older adults increasingly prioritize emotional meaning. Reflecting this shift, prosocial behavior such as charitable giving tends to rise with age. Yet this pattern has not been tested in large, nationally representative surveys. This gap is important given that older adults hold a majority of national wealth and their giving decisions carry broad societal implications. Using data from the Federal Reserve’s Survey of Consumer Finances, we found that in 2022, adults aged 55 and over held 73% of the national wealth (ages 40-54: 20%; under 40: 7%), up from 53% in 1989, a one-third surge across three decades. Prosocial behavior was examined in the Panel Study on Income Dynamics, a nationally representative sample collected in 2023 (N = 7,694; ages 18–98, M = 48, SD = 16). Consistent with prior findings, older adults were more likely to donate (β = 0.01, p<.001, R²=.081) and donated larger amounts (β = 38.47, p<.001, R²=.013) than younger adults. Similarly, older adults were more likely to volunteer (β = 0.002, p<.001, R²=.005) and volunteered more hours (β = 0.01, p<.001, R²=.004) than younger adults. These effects held after controlling for wealth, race, and marital status. Taken together, the growing concentration of wealth among older adults and their heightened prosociality suggest they may represent a strategic focus for philanthropy. Encouraging charitable giving in later life could help redirect intergenerational wealth transfers to promote equity.

